# The Relationships among Tryptophan, Kynurenine, Indoleamine 2,3-Dioxygenase, Depression, and Neuropsychological Performance

**DOI:** 10.3389/fpsyg.2017.01561

**Published:** 2017-10-04

**Authors:** Knut A. Hestad, Knut Engedal, Jon E. Whist, Per G. Farup

**Affiliations:** ^1^Department of Research, Innlandet Hospital Trust, Brumunddal, Norway; ^2^Department of Psychology, Faculty of Social Sciences and Technology Management, Norwegian University of Science and Technology, Trondheim, Norway; ^3^Department of Public Health, Inland Norway University of Applied Sciences, Elverum, Norway; ^4^Norwegian Center for Aging and Health, Vestfold Health Trust, Tønsberg, Norway; ^5^Department of Medical Biochemistry, Innlandet Hospital Trust, Brumunddal, Norway; ^6^Unit for Applied Clinical Research, Department of Cancer Research and Molecular Medicine, Faculty of Medicine, Norwegian University of Science and Technology, Trondheim, Norway

**Keywords:** tryptophan, kynurenine, indoleamine 2,3-dioxygenase, IDO, depression, neuropsychology

## Abstract

It has been suggested that the metabolic enzyme indoleamine 2,3-dioxygenase (IDO) is a biological mediator of inflammation related to the psychopathology of depression, with a Kynurenine (KYN) increase in the Tryptophan (TRP) metabolic pathway, resulting in reduced Serotonin. In this study, we examined KYN, TRP, and the ratio of KYN to TRP concentrations × 10^3^ (KT Ratio) in serum and cerebrospinal fluid (CSF) in (a) a group of depressed patients and (b) a control group of patients referred to a neurologic outpatient clinic for whom no specific diagnosis could be established. The KT Ratio is considered an index that represents IDO. The participants were examined with the Beck Depression Inventory II (BDI-II), the Montgomery Aasberg Depression Rating Scale (MADRS), and a neuropsychological test battery. We found no significant differences between the two study groups with respect to TRP, KYN, or KT Ratio in serum or CSF. Differences in neuropsychological performance between the two patient groups could be seen in the following tests: Animal Fluency, Digit Symbol, the DKEFS Color-Interference Test (Naming Part), Trail Making Test A and B, and the Grooved Pegboard Non-dominant Hand. KYN in serum correlated highly with KYN in CSF. KYN in serum correlated significantly with both age and gender. When analyzing males and females separately, we found that women had a lower level of TRP in both serum (Mann–Whitney *U*-test: TRP in Serum; *p* = 0.001) and CSF (Mann–Whitney *U*-test: TRP in CSF; *p* = 0.003). Women had a lower level of KYN in serum (*p* = 0.029) than men did. Age was positively associated with KYN. KYN in CSF correlated only with age, however; there were no gender differences. No significant relationship was seen between BDI-II and MADRS on the one hand, and KYN and TRP on the other. KYN in CSF as the KT Ratio in both serum and CSF was associated with neuropsychological performance. Thus, we suggest that KYN and KT Ratio are related more strongly to neuropsychological performance than to affective symptoms in depression.

## Introduction

Depression is the world’s most widespread mental disorder, affecting over 350 million individuals (WHO, 2012). The mechanisms thought to be associated with depression are (a) signaling through neurotransmitters and (b) the axis of the hypothalamus, pituitary and adrenal cortex, with (c) a more recent focus on inflammation. These pathways, interacting with each other, provide a biological basis for the development of depression, and elevation of proinflammatory cytokines and other signs of inflammation are frequently seen (Hestad et al., 2003; Young et al., 2014; Poon et al., 2015). Examining gene expression in major depression showed a significantly different regulation of inflammatory/immune pathways than exhibited by healthy controls (Jansen et al., 2015). And finally, in patients who received pharmacological treatment with cytokines or other immune modifiers, about 1/3 developed severe depression (Dantzer et al., 1999; Capuron et al., 2002a,b,c). Yet, several other patient groups that may be at least partly affected by psychological factors—fatigue, myalgic encephalopathy, and schizophrenia, for example—have also shown a rise in pro-inflammatory cytokines. Low-grade inflammation seems to be a specific biomarker related to depression, however. To examine this possibility, it would be of interest to examine a similar patient control group with many of the same symptoms, but with no signs of overt depression. We did discuss the addition of a control group of healthy individuals, but did not seriously consider it, given the requirement of a spinal tap and the possible side effects it represents.

It has been suggested that the metabolic enzyme indoleamine 2,3-dioxygenase (IDO) is a biological mediator of inflammation related to depressive disorders, as seen in animal models and after drug therapy in humans with interferon-α (O’Connor et al., 2009a,b; Raison et al., 2010). The effects on depression are via IDO and Tryptophan-2,3-dioxygenase (TDO), with an increase in Kynurenine (KYN) in the Tryptophan (TRP) metabolic pathway, resulting in a reduced amount of Serotonin. Many proinflammatory cytokines, such as IL-1, IL-2, IL-6, and IFN-γ can induce IDO and TDO. TRP is the substrate for IDO and TDO, and the metabolized product is KYN (Guillemin et al., 2001). With inflammation, and as levels of IDO and TDO increase, an increase in TRP will be used by IDO and TDO in the KYN production. The result will be reduced TRP as the basis for Serotonin production. As IDO and TDO increase, there will be less production of Serotonin, which is associated with depression (Hayaishi, 1996; Stone and Darlington, 2002; Wirleitner et al., 2003; Schiepers et al., 2005). It has been shown that the serum KYN-to-TRP Ratio (KYN/TRP × 10^3^) KT Ratio is a reliable indication of IDO (Schrocksnadel et al., 2006; Raison et al., 2010). Because depression is linked to such monoamines as Serotonin and Noradrenaline, there may be less regulation in neurotransmission related to these neurotransmitters in depression, with increased production of KYN (Schildkraut, 1965; Ressler and Nemeroff, 1999, 2000; Dursun et al., 2001; Bonaccorso et al., 2002; Capuron et al., 2002c, 2003; Castanon et al., 2002; Wichers and Maes, 2004; Muller and Schwarz, 2006). In addition, IDO and TDO may activate depression through the metabolite 3-hydroxy-kynerunine (3OH-KYN) and quinolinic acid (QUIN) (Heyes et al., 1996; Guillemin et al., 2005). 3OH-KYN and QUIN are probably involved in a number of neurodegenerative disorders, like Parkinson’s Disease and HIV-dementia (Heyes et al., 1992; Stone, 2001; Wichers and Maes, 2004). Both 3OH-KYN and QUIN are neurotoxic, and it has been proposed that QUIN can create atrophy of the hippocampus and a reduction of corticosteroid receptors (Bremner et al., 2000; Wichers and Maes, 2004).

Although several studies have examined plasma or serum levels of inflammatory markers in psychopathology of depressive disorders, data on Tryptophan, Kynurenine, Indoleamine 2,3-dioxygenase in both serum and cerebrospinal fluid (CSF) have rarely been studied. A PubMed search with the words Tryptophan, Kynurenine, serum, CSF resulted in 21 studies, none of which were related to depression. We wanted to rectify this omission by examining these variables in a depressive group and a control group of patients with no depression. In addition, we asked: ‘How are serum and CSF levels connected?’

We hypothesized that patients with a diagnosis of depression would present with a specific and different serum and CSF, KYN, and TRP profile than would patients with no depression. In particular, we expected to find higher levels of KYN and KT Ratio in both serum and CSF among depressed patients than among non-depressed patients. We therefore examined two patient groups: one with depression and one without. The non-depressed group, which served as a control group, was referred to a neurological clinic because of diffuse (neurological) symptoms such as fatigue, but no diagnosis could be established. Because a recent study has demonstrated that IDO is related to depressive symptoms more strongly in women than in men (Elovainio et al., 2012), we analyzed TRP, KYN, and KT Ratio separately for males and females.

Cognitive dysfunction is common in depression (Veiel, 1997; Gualtieri et al., 2006; Farup and Hestad, 2015). It is not known, however, whether it is part of the development of affective symptoms in depression or if two separate developments and biological pathways are involved. We therefore posed two research questions.

(1) Does the concentration of TRP, KYN, and KT Ratio in serum and CSF differ between patients with depression and a similar patient group without depression? And as a secondary aim, do patients with depression differ in cognitive performance compared to patient group with no depression?
(2) Are TRP and/or KYN and KT Ratio concentrations in serum and CSF associated with affective symptoms and/or cognitive performance?

## Materials and Methods

### Patients and Controls

A group of 49 depressed patients were recruited from inpatient wards and outpatient clinics of the Mental Health Unit at Innlandet Hospital Trust, Norway. They were all over 18 years of age and were diagnosed with depression using (a) the International Statistical Classification of Diseases and Related Health Problems, 10th revision [ICD-10], F32–34 spectra; and (b) DSM-IV^TR^, mood disorders. For participants in the depression group, it was decided that brain imaging should be conducted if there were any indication or suspicion of cerebral disorder. None of the depressed patients showed any indication or suspicion of such a disorder, so they were not examined with brain imaging.

Thirteen of the 49 depressive patients were using a combination of anti-depressive and antipsychotic medication; 22 were medicated with anti-depressives only and 3 with only antipsychotic medication.

The control group comprised 31 patients over 18 years of age with neurological symptoms who were referred to the Department of Neurology at the same hospital for a thorough investigation. The inclusion criteria were no disease and no neurological sign of disease. None of the patients had neurological signs of disease; and none of their laboratory tests indicated cerebral disorder. Clinically, they had many similar symptoms seen in depressed patients, including tiredness, fatigue, lack of initiative, and the feeling that the body is not functioning. They had no overt signs of depression, however: mood changes or emotional state. Other than depression, the most common symptom was fatigue, as reported by 45 of the depressed patients and 25 of the controls. Nineteen of the control patients had a neuropsychological assessment and were included in the study. Of these 19 controls, 14 reported fatigue. Lack of funding prevented our giving 12 patients from the control group a neuropsychological examination when they were recruited.

All patients were recruited consecutively from either the psychiatric or neurological ward after the psychiatrists or neurologists, who were informed about the study, determined that the patients met the criteria for inclusion in the study.

### Examinations and Assessments

A medical history was recorded, a routine clinical examination performed, and hematological and biochemical screening tests conducted on all participants. Peripheral blood was collected, allowed to clot for 30 min before 10 min of centrifugation at 2000 *g*. CSF samples were obtained by a trained neurologist and centrifuged within 30 min at 2000 *g* for 10 min. Serum and CSF was aliquoted and stored at -70°C until analyses were performed. All blood samples were taken between 08.30 and 10.00. The spinal tap was done before 12.00 noon, except for two depressed patients for whom the procedure was performed closer to 13.00. The patients were not required to fast before blood or CSF sampling.

A neurologist conducted a detailed examination of the patients, including a routine examination of CSF, in the Department of Neurology.

TRP and KYN levels in serum and CSF were determined by high-performance liquid chromatography (Widner et al., 1997). In brief, an aliquot of 200 μL serum was precipitated with 125 μl 25% (w/v) trichloroacetic acid, mixed, and centrifuged. For separation, reversed-phase Kinetex columns (2.6 μ C18 100A) from Phenomenex (Torrance, CA, United States) were used. KYN was detected with an ultraviolet absorption detector, and TRP was detected using a fluorescence detector. Instrumental analysis was performed on Agilent Infinity 1290 (Agilent Technologies, Santa Clara, CA, United States). The KT Ratio was calculated and used as a measure of tryptophan degradation and as IDO indicator.

All patients were examined independently with two depression scales. (a) Montgomery Aasberg Depression Rating Scale (MADRS) (Montgomery and Asberg, 1979) yielded four categories: no depression 0–6, mild depression 7–19, moderate depression 20–34, and severe depression > 34. (b) Beck Depression Inventory II (BDI-II) (Beck et al., 1996; Steer et al., 1997, 1999) also yielded four categories: minimal depression 0–13, mild depression 14–19, moderate depression 20–28, and severe depression 29–63. The patients were examined with a neuropsychological test battery of eight measures assessing cognitive domains that are commonly involved in depression.

• Mini-Mental State Examination (MMSE) is a test for global cognitive function (Folstein et al., 1975).
• Trail Making Test A (simple); Trail Making Test B (complex): the Trail Making Test measures attention, visual searching, mental processing speed, set shifting, and cognitive flexibility (Strauss et al., 2006).
• Grooved Pegboard Test, a test of fine motor control: this test measures response time in seconds with dominant and non-dominant hand (Kløve, 1974).
• Hopkins Verbal Learning Test-R (HVLT): immediate total recall and delayed recall (Brandt, 1991, 2001).
• Brief Visual Memory Test-R (BVMT) measures immediate and delayed visual recall (Benedict et al., 1998).
• Wechsler Adult Intelligence Scale, 3rd Edition (WAIS-III), the subtests of which are Digit Symbol and Symbol Search measures processing speed, visual perception, attention and concentration, motor and mental speed (Wechsler et al., 2003).
• The D-KEFS Color Word Interference Test: Test 1, color naming; Test 2, color name reading; Test 3 inhibition; Test 4 inhibition/switching. The test measures the Stroop effect, attention, cognitive processing, and mental stimulus control (Strauss et al., 2006).
• Controlled Oral Word Association Test (COWAT), which measures the ability to produce words within 1 min: Words (number of Words on F, A, and S), Clothes (number of clothes), Animals (number of Animals named) (Strauss et al., 2006).

### Ethics Statement

The study was reviewed and approved by the Regional Committees for Medical and Health Research Ethics, South East A, Reference No. 2009/2196a, and all participants provided a written informed consent form before they were included in the study.

### Statistics

Independent Mann–Whitney *U*-test or chi-square analyses were performed between the two groups to test group differences in age, education, gender, KYN, TRP, KT Ratio, depressive symptom forms, and neuropsychological test results. Correlations among KYN, TRP, and KT Ratio, age, and gender were assessed with Spearman’s rho. Linear multiple regression was used to study independent predictors. Statistical significance was set at *p* < 0.05. SPSS Version 24 (IBM Corporation, Armonk, NY, United States) was used for the analyses.

## Results

Clinical examination, CSF examination, computed tomography, or magnetic resonance imaging revealed no cerebral neuropathology in any of the patients (The computed tomography or magnetic resonance imaging was undertaken for all participants in the neurological group). Controlling for age, we analyzed our data to see if the anti-depressive drugs or the combination of anti-depressive and anti-psychotic drugs had had an impact on the test results in the depression group. No significant results were found (data not shown).

Participant characteristics with comparisons between the two study groups are shown in **Table 1**. There were no significant differences with respect to TRP, KYN, or KT Ratio in serum or CSF. When we analyzed men and women separately independent of depression, however, women were found to have a lower level of TRP in both serum (Mann–Whitney *U*-test: TRP in serum; *p* = 0.001) and CSF (Mann–Whitney *U*-test: TRP in CSF; *p* = 0.003) than men. Women also had a lower level of KYN in serum (*p* = 0.029), but not CSF (*p* = 0.15). No differences were found for KT Ratio (data not shown). When the analyses were broken down for men and women in the depression and the control group separately, the same results were found in the depression group for TRP in serum (*p* = 0.035) and CSF (*p* = 0.04) and for the neurological group for TRP in serum (*p* = 0.018) and CSF (*p* = 0.006). The KYN serum difference between women and men was not significantly different in either the depression or control group (data not shown).

**Table 1:** Demographic, biological, and neuropsychological group differences in the study groups as mean (SD) or exact numbers.

	Neurological group *N* = 31 Mean (*SD*)	Depression group *N* = 44 Mean (*SD*)	*p*-value, Mann–Whitney *U* [univariate analysis corrected for age and gender]
Age	44.10 (13.32)	44.66 (15.06)	0.52
Gender (women/men)	22/9	23/21	
Education in years	13.53 (2.36)	12.55 (2.84)	0.11
Kynurenine serum (μM)	1.99 (0.511)	2.17 (0.525)	0.06 [0.12]
Tryptophan serum (μM)	54.68 (10.80)	57.85 (12.39)	0.46 [0.73]
K/T ratio × 1000 serum	37.48 (10.74)	38.53 (10.13)	0.50 [0.23]
Kynurenine CSF (μM)	0.0616 (0.018)	0.0661 (0.027)	0.71 [0.24]
Tryptophan CSF (μM)	2.74 (2.02)	6.43 (21.60)	0.78 [0.35]
K/T ratio × 1000 CSF (μM)	27.38 (12.67)	27.34 (12.23)	0.53 [0.53]
	*N* = 29	*N* = 44	
Beck depression scale-II	6.5 (6.7)	30.1 (12.4)	**<0.001 [<0.001]**
	*N* = 19	*N* = 44	
Montgomery Aasberg depression scale	8.1 (5.9)	28.00.(8.03)	**<0.001 [<0.005]**
Mini-mental state examination	29.2 (1.0)	28.3 (1.8)	0.08 **[0.05]**
HVLT immediate total recall	23.7 (5.2)	22.6 (4.9)	0.52 [0.38]
HVLT delayed recall	9.2 (2.4)	8.3 (2.7)	0.28 [0.26]
BVMT immediate total recall	20.7 (5.4)	19.7 (7.1)	0.69 [0.56]
BVMT delayed recall	8.6 (2.3)	7.7 (2.8)	0.19 [0.11]
Trail making test A	40.4 (27.7)	51.6 (24.0)	**0.011 [0.03]**
Trail making test B	79.4 (32.8)	121.9 (78.6)	**0.025 [0.005]**
Grooved pegboard test (dominant hand)	72.2 (15.1)	89.4 (41.1)	0.11 **[0.01]**
Grooved pegboard test (non-dominant hand)	80.8 (22.1)	98.0 (35.1)	**0.02 [0.01]**
WAIS-III digit symbol	60.3 (15.2)	51.5 (17.3)	**0.046 [0.006]**
WAIS-III symbol search	28.1 (8.7)	25.2 (8.3)	0.22 **[0.02]**


As expected, the depression and diffuse neurological symptom groups differed significantly on both Beck-II and MADRS. Differences in neuropsychological performance could be seen in Animal fluency, WAIS-III Digit symbol, The D-KEFS color naming, Trail Making Tests A and B, and the Grooved Pegboard non-dominant hand, with best performance in the neurological group. There results were basically the same when controlled for age and gender.

**Table 2** presents correlations among KYN, TRP, and the KT Ratio in serum and CSF by age and gender. KYN concentrations in serum and CSF correlated significantly with each other; this was not the case with TRP. Age was positively correlated with KYN levels, but KYN in serum correlated positively with age only for men. On the other hand, KYN in CSF correlated positively with age, but there were no gender differences. Regarding TRP, the correlations with age and gender were weaker, but in the same direction as for KYN. TRP in serum was significant only for gender, whereas TRP in CSF was related to both age and gender. KT Ratio in CSF and serum correlated highly with each other, and showed also positive correlations with age but not with gender.

**Table 2:** Correlations among kynurenine, tryptophan kynurenine/tryptophan in CSF and serum correlated (rho), by age and gender.

	Kynurenine CSF (μM)	Tryptophan CSF (μM)	Kynurenine/Tryptophan CSF	Kynurenine serum (μM)	Tryptophan serum (μM)	Kynurenine/Tryptophan serum	Age	Gender (women/men)
Kynurenine CSF (μM)		**0.522**	**0.496**	**0.614**	-0.003	**0.576**	**0.471**	0.168
		***p* < 0.001**	***p* < 0.001**	***p* < 0.001**	*p* = 0.982	***p* < 0.001**	***p* < 0.001**	*p* = 0.150
Tryptophan CSF (μM)			**-0.356**	0.162	0.207	-0.001	**0.268**	**0.351**
			***p* = 0.002**	*p* = 0.166	*p* = 0.074	*p* = 0.993	***p* = 0.020**	***p* = 0.002**
Kynurenine/Tryptophan CSF				**0.584**	-0.136	**0.650**	**0.309**	-0.035
				***p* < 0.001**	*p* = 0.244	***p* < 0.001**	***p* = 0.007**	*p* = 0.764
Kynurenine serum (μM)					**0.298**	**0.682**	**0.470**	**0.254**
					***p* = 0.009**	***p* < 0.001**	***p* < 0.001**	**>*p* = 0.028**
Tryptophan serum (μM)						-**0.423**	0.022	**0.383**
						***p* < 0.001**	*p* = 0.849	***p* = 0.001**
Kynurenine/Tryptophan serum							**0.448**	-0.063
							***p* < 0.001**	*p* = 0.592
Age								0.138
								*p* = 0.218
Gender (women/men)								


*Kynurenine in serum and CSF is highly correlated with each other. Kynurenine in CSF is significantly correlated with age, but not with gender. Kynurenine in serum is significantly correlated with age and gender. Tryptophan in serum is significantly correlated with gender but not age. Men have higher values on both kynurenine and tryptophan in serum. Thus, only Kynurenine seems to be related to age. Only serum values of Kynurenine and Tryptophan are significantly related to gender. Kynurenine/tryptophan in CSF and serum correlated highly with each other, and showed also positive correlations with age but not with gender.*

**Table 3** presents correlations of TRP, KYN, and the KT Ratio to predict scores on BDI-II, MADRS, and neuropsychological tests in linear regressions, controlling for age, gender, and group status (depression or neurological group). Because of their distributions, Trail Making Test A and B, Grooved Pegboard Test, and inhibition of word and color in the D-KEFS inhibition were log transformed. No significant correlations were found between BDI-II/MADRS on the one hand and KYN, TRP or KT Ratio on the other.

**Table 3:** The table shows linear regressions with the neuropsychological tests as dependent variables.

	Kynurenine serum, partial correlations and (*p*-values)	Kynurenine spinal fluid, partial correlations and (*p*-values)	Tryptophan serum, partial correlation and (*p*-values)	Trypophan spinal fluid, partial correlations and (*p*-values)	KT Ratio serum, partial correlations and (*p*-values)	KT Ratio spinal fluid, partial correlation and (*p*-values)
BDI-II	0.07 (0.58)	0.03 (0.73)	-0.008 (0.95)	-0.10 (0.41)	0.08 (0.50)	0.08 (0.50)
MADRS	0.13 (0.32)	0.02 (0.88)	-0.02 (0.87)	-0.15 (0.38)	0.14 (0.30)	0.09 (0.50)
MMSE	-0.068 (0.61)	**-0.29 (0.028)**	0.018 (0.89)	0.06 (0.66)	-0.11 (0.39)	-0.16 (0.22)
HVLT learning	-0.20 (0.13)	-0.08 (0.57)	0.12 (0.38)	0.00 (1.00)	**-0.28 (0.03)**	-0.13 (0.33)
HVLT delayed recall	-0.21 (0.16)	-0.19 (0.15)	0.09 (0.52)	-0.03 (0.87)	**-0.28 (0.03)**	-0.17 (0.19)
BVMT learning	-0.22 (0.09)	-0.12 (0.37)	0.002 (0.99)	0.00 (0.96)	-0.18 (0.18)	-0.09 (0.48)
BVMT delayed recall	-0.21 (0.11)	-0.17 (0.20)	-0.05 (0.75)	-0.03 (0.85)	-0.14 (0.27)	-0.08 (0.56)
Trail making test part A	0.11 (0.40)	0.15 (0.25)	-0.25 (0.06)	0.11 (0.39)	**0.30 (0.02)**	0.29 (0.10)
Trail making test part B	0.06 (0.67)	0.13 (0.32)	0.04 (0.74)	0.11 (0.43)	0.11 (0.41)	0.09 (0.49)
Grooved pegboard DH	0.03 (0.85)	**0.33 (0.009)**	-0.03 (0.54)	0.08 (0.53)	0.08 (0.55)	0.15 (0.26)
Grooved pegboard NDH	0.11 (0.41)	** 0.31 (0.015)**	-0.04 (0.79)	0.12 (0.37)	0.11 (0.42)	0.07 (0.61)
Digit symbol WAIS-III	-0.09 (0.51)	**-0.26 (0.04)**	-0.11 (0.41)	-0.02 (0.90)	-0.17 (0.20)	-0.13 (0.31)
Symbol search WAIS-III	-0.08 (0.55)	-0.17 (0.21)	0.15 (0.26)	-0.12 (0.35)	-0.16 (0.23)	-0.18 (0.16)
D-KEFS color naming	0.09 (0.51)	**0.26 (0.05)**	-0.07 (0.57)	-0.03 (0.85)	0.20 (0.12)	**0.27 (0.04)**
D-KEFS color reading	0.07 (0.60)	0.14 (0.30)	-0.04 (0.77)	0.04 (0.78)	0.13 (0.31)	0.21 (0.11)
D-KEFS inhibition	-0.03 (0.83)	0.15 (0.27)	-0.14 (0.28)	0.08 (0.56)	0.06 (0.65)	0.21 (0.11)
D-KEFS inhibition/switching	0.22 (0.09)	0.21 (0.10)	-0.14 (0.28)	-0.00 (0.98)	**0.30 (0.02)**	**0.37 (0.004)**
Verbal fluency FAS	-0.17 (0.38)	-0.04 (0.77)	-0.02 (0.88)	-0.15 (0.25)	-0.14 (0.28)	0.05 (0.68)
COWA cloths	-0.22 (0.10)	-0.02 (0.21)	-0.00 (0.99)	-0.04 (0.75)	-0.22 (0.10)	0.02 (0.89)
COWA animals	-0.02 (0.88)	0.06 (0.68)	0.12 (0.37)	-0.15 (0.27)	0.04 (0.76)	0.23 (0.07)


*Independent variables: kynurenine, tryptophan, and the kynurenine/tryptophan ratio (KT Ratio). Age, gender, and group membership as covariates. Partial correlation and (*p*-values) are presented.*

As can be seen in **Table 3**, KYN in CSF correlated with neuropsychological performance when age, gender, and group membership were controlled. Further associations were seen when the KT Ratios were examined. No such correlations were found for TRP, and no significant associations were found between BDI-II, MADRS on the one hand and KYN, TRP, or KT Ratio on the other. When the same tests were performed without controlling for group status, however, the partial correlations between KYN in CSF and KT Ratios relative to the neuropsychological test performance were stronger.

• KYN in CSF: MMSE, partial correlation -0.32, *p* = 0.013; Grooved Pegboard dominant hand (Log values), partial correlation = 0.34, *p* = 0.007; Grooved Pegboard non-dominant hand (Log values), partial correlation = 0.31, *p* = 0.017; Digit Symbol WAIS-III, partial correlation = -0.30, *p* = 0.017, D-KEFS color naming, partial correlation = 0.31, *p* = 0.016.
• KT Ratio in serum: HVLT-learning, partial correlation -0.294, *p* = 0.022; HVLT-delayed recall, partial correlation -0.301, *p* = 0.018; Log Trail Making Test Part A, partial correlation 0.34, *p* = 0.008; D-KEFS color word inhibition/switching, partial correlation = 0.32, *p* = 0.012.
• KT Ratio in CSF: D-KEFS color naming, partial correlation = 0.28, *p* = 0.029, DKEFS inhibition/switching, partial correlation = 0.37, *p* = 0.003.

## Discussion

We are unaware of any other study that compared a depression group to a similar patient group, as we have done in this study with regard to KYN, TRP, and KT Ratio in serum and CSF.

No differences in KYN, TRP, or KT Ratio concentrations in serum and CSF were found between the two patient groups. Women, however, had lower levels of TRP in both serum and CSF. Because women in general have a higher incidence of depression, we propose that this difference may be of importance with some parts of the development of depression. Elovainio et al. (2012) found that the KT Ratio in serum (as a marker of IDO) predicted depressive symptoms in women only (*p* = 0.03), even after adjustment for baseline depression scores. In a study of young adult (24–39 years) Finns, it was found that the IDO enzyme is involved in immune regulation of early atherosclerosis, particularly among females, and could constitute a marker of immune activation for atherosclerosis in females (Pertovaara et al., 2007). In examining TRP and KYN in men and women, however, we found these markers to be higher in men, and no gender differences were found in KT Ratio in serum or CSF. There were relatively strong correlations for KYN, TRP, and KT Ratio concentrations with both age and gender (older people and men), particularly for KYN and KT Ratio relative to age. We are aware of no other study that has demonstrated these findings regarding CSF in depressed patients. This finding could indicate the involvement of other biological mechanisms as people age (Alexopoulos, 2005). Depression in old people is often seen in individuals with co-morbid physical disorders, which increases the complexity of the biological mechanisms of depression (Alexopoulos, 2005).

KYN concentrations (and KT Ratios) in serum and CSF correlated significantly with each other. TRP in serum and CSF showed no significant correlation. Theoretically, Interferon-γ will enhance TRP metabolism by inducing IDO and thereby catalyze the first step of TRP degradation, forming the KYN. Our results could not support Interferon-γ as a specific trait in depression, however. In addition, when we studied cytokines in serum and CSF, as reported in another paper (Hestad et al., 2016), we found no significant correlation between INF-γ in serum and CSF and depression.

Although the two groups were similar with respect to clinical symptoms, there was a clear trend toward differences in cognitive performance between the depression and control groups. Depression in itself, therefore, seems to be responsible for the depression group’s inferior cognitive performance.

KYN in CSF was related to some degree to neuropsychological performance. Poorer performance on MMSE, Grooved Pegboard, Digit Symbol WAIS-III, and D-KEFS Color all correlated with KYN in CSF. The partial correlations for these tests were modest—all below 0.4. More associations were seen with the KT Ratio, however. HVLT learning and delayed recall and the Trail Making Test Part A were all significantly associated with KT Ratio in serum, and this finding seems to be partly independent of depression. Higher levels of the KT Ratio indicated poorer performance on the neuropsychological tests. The KT Ratio in CSF showed significant associations with D-KEFS color naming and D-KEFS inhibiting/switching. These findings could indicate support for the theory that too much KYN at the expense of Serotonin results in metabolites that influence cognitive performance (Wichers and Maes, 2004). Thus KYN in CSF and KT Ratio in both serum and CSF may be related to some degree to cognitive performance in the psychopathology of depression but not to affective symptoms as measured with MADRS and BDI-II. Because all the analyses were controlled for age, gender, and group membership, the findings are independent of these factors. KYN in CSF or KT Ratio may not explain all the variance in neuropsychological performance. When the regression analyses were performed without controlling for depression, the associations were stronger, but in the same direction. We therefore suggest that KYN in CSF and KT Ratio in both serum and CSF are related to cognitive performance, as measured by the neuropsychological tests, but not to depression, as measured by BDI-II or MADRS. One consideration is the effect of the variables that are being partialled out: When it is large, the resulting statistics can be unstable, especially in model samples. Nevertheless, without controlling for these factors, the findings went in the same direction for the KYN, TRP, and KT Ratios regarding depression and cognitive performance.

## Conclusion

No significant differences were found between a group of depressed patients and controls regarding KYN, TRP, KT Ratios in serum and CSF. Women seem to have a lower level of TRP in both serum and CSF than men do. There was a clear trend toward inferior neuropsychological performance in the depression group compared to the controls. KYN, TRP, and KT Ratio are strongly related to age and gender in the two samples, independent of depression. In the original studies of KYN and TRP related to medication with Interferon-α, the vegetative or sickness symptoms emerged weeks before depression (Capuron et al., 2002a,c, 2004). The symptoms pointed out are malaise, lassitude, fatigue, numbness, coldness, muscle and joint aches, and reduced appetite. Thus, KYN and KT Ratio could be related more strongly to sickness than to affective symptoms, manifested in our study as poorer neuropsychological performance. The cognitive symptoms were related to KYN in CSF and KT Ratio in both serum and CSF. This was not so for the affective symptoms, which seem to be independent of both TRP and KYN. Low-grade inflammation related to KYN and TRP with the pathway of IDO in serum or plasma has been shown to be related to depression. It may be that this relationship relates in turn not to the affective symptoms, but to cognitive impairment. We suggest that a rise in inflammatory markers in depression is an indicator of general malfunction and that anti-depressive drugs, ECT, and physical activity may help alleviate these symptoms. It is suggested that the mechanism behind it is through treatment of activated inflammation, and that there is a close connection between body and mind. Treat the body, and it will be easier to treat the mind—the depressive symptoms. We believe that it will be necessary to study the interaction between body and mind in the treatment of depression and other similar disorders such as tiredness and fatigue. For the future it would be interesting to examine whether KYN, TRP and the ratio of KT are correlated with other variables in depression, such as treatment resistance, depression duration, number of episodes, and not least, sickness behavior, with such factors as malaise, lassitude, fatigue, numbness, muscle and joint aches, and changes in appetite.

## Author Contributions

KH designed the study and did the writing of the manuscript. KE took part in the planning of the study and contributed to the writing process. JW took part in the planning of the study and contributed to the writing process. PF took part in the planning of the study and contributed to the writing process.

## Conflict of Interest Statement

The authors declare that the research was conducted in the absence of any commercial or financial relationships that could be construed as a potential conflict of interest.
